# Supplementary material for: Experience of clinical services shapes attitudes to mental health data sharing: findings from a UK-wide survey
Source: BMC Public Health. 2022 Feb 19;22:357. doi: 10.1186/s12889-022-12694-z (PMC8858475; doi:10.1186/s12889-022-12694-z)
Supplement: Supplementary file 1 — Additional file 1. [file 12889_2022_12694_MOESM1_ESM.docx]

**Supplementary material 1:** Questions and original response options presented to participants

Q1.4 Have you ever used the UK National Health Service (NHS), for any reason?

- Yes (1)
- No (2)

Skip To: End of Survey If Have you ever used the UK National Health Service (NHS), for any reason? = No

End of Block: Information and consent

Start of Block: Data sharing

Q2.1
This survey will ask you some questions about **DATA** and **RESEARCH**.
 
**Data** means information about you. **Health data** is normally collected by doctors and other medical professionals. Examples of health data might include your weight at birth, your blood pressure, and your GP's notes about your health.
 
**Research** means finding out new things. **Health research** is normally carried out by universities, often in partnership with hospitals and sometimes with companies that make medicines and medical devices (e.g. pacemakers). Examples of health research might include looking at the long-term effects of taking vitamin supplements, or understanding how having a parent with dementia changes your chances of also having dementia.

Q2.2
Would you be willing to share your health data for research purposes?

- Yes (1)
- No (2)

| Page Break |  |
| --- | --- |

Q2.3
**Physical** health data might include medical images (like X-rays), blood test results, or information about long-term conditions (e.g. whether you have diabetes or not).
 
**Mental** health data often involves the same types of things. For example it might include brain scan images, or information about long-term conditions (e.g. whether you have ever had a diagnosis of depression).

Q2.4 Would you be willing to share your **physical** health data for research purposes?

- Yes (1)
- No (2)

Q2.5 Would you be willing to share your **mental** health data for research purposes?

- Yes (1)
- No (2)

| Page Break |  |
| --- | --- |

Q2.6 Thinking about data more generally (not only health data), how likely would you be to share the following types of data for research purposes?

|  | Extremely unlikely (1) | Somewhat unlikely (2) | Neither likely nor unlikely (3) | Somewhat likely (4) | Extremely likely (5) |
| --- | --- | --- | --- | --- | --- |
| Social media posts (1) |  |  |  |  |  |
| Physical health data (2) |  |  |  |  |  |
| Financial information (e.g. credit rating) (3) |  |  |  |  |  |
| Mental health data (4) |  |  |  |  |  |
| Employment history (5) |  |  |  |  |  |
| Ethnicity (9) |  |  |  |  |  |
| Marital status (10) |  |  |  |  |  |

| Page Break |  |
| --- | --- |

Q2.7 How likely would you be to share the following types of **physical** health data for research purposes?

|  | Extremely unlikely (1) | Somewhat unlikely (2) | Neither likely nor unlikely (3) | Somewhat likely (4) | Extremely likely (5) |
| --- | --- | --- | --- | --- | --- |
| Weight (1) |  |  |  |  |  |
| Height (2) |  |  |  |  |  |
| Diagnosis (3) |  |  |  |  |  |
| Test results (e.g. blood tests) (4) |  |  |  |  |  |
| Genetic information (5) |  |  |  |  |  |
| GP notes (6) |  |  |  |  |  |
| Records of which services you have used (e.g. GP, pain management, rheumatology, cancer services) (7) |  |  |  |  |  |
| Medical images (e.g. X-rays, ultrasound) (8) |  |  |  |  |  |

| Page Break |  |
| --- | --- |

Q2.8 How likely would you be to share the following types of **mental** health data for research purposes?
 

*Please note we are interested in your views even if you do not have a mental health condition.*

|  | Extremely unlikely (1) | Somewhat unlikely (2) | Neither likely nor unlikely (3) | Somewhat likely (4) | Extremely likely (5) |
| --- | --- | --- | --- | --- | --- |
| Diagnosis (1) |  |  |  |  |  |
| Scores on mental health assessment questionnaires (2) |  |  |  |  |  |
| Genetic information (3) |  |  |  |  |  |
| GP notes (4) |  |  |  |  |  |
| Records of which services you have used (e.g. GP, eating disorder clinic, psychiatrist) (5) |  |  |  |  |  |
| Medical images (e.g. MRI scan) (6) |  |  |  |  |  |
| Therapist/psychologist/psychiatrist's notes (7) |  |  |  |  |  |

| Page Break |  |
| --- | --- |

Q2.9 In your opinion, how trustworthy are the following organisations when it comes to storing and using **mental** health data for research purposes?

|  | Not at all trustworthy    1 (1) | 2 (2) | 3 (3) | 4 (4) | Very trustworthy    5 (5) |
| --- | --- | --- | --- | --- | --- |
| The NHS (1) |  |  |  |  |  |
| The UK government (2) |  |  |  |  |  |
| Your local authority/council (3) |  |  |  |  |  |
| Universities (4) |  |  |  |  |  |
| Mental health charities (5) |  |  |  |  |  |
| Private companies (6) |  |  |  |  |  |
| Devolved governments (e.g. Scottish, Welsh or Northern Irish governments) (7) |  |  |  |  |  |

| Page Break |  |
| --- | --- |

Q2.10 How would the following measures change the likelihood that you would be willing to share your **mental** health data for research purposes?

|  | Much less likely (1) | Less likely (2) | No change (3) | More likely (4) | Much more likely (5) |
| --- | --- | --- | --- | --- | --- |
| I would be asked for permission every time someone wanted to look at my data. (1) |  |  |  |  |  |
| My data would be part of a huge database containing data from many hundreds of other people. (2) |  |  |  |  |  |
| I would have no control over what my data was used for in the future. (8) |  |  |  |  |  |
| My name would be removed from all data used for research purposes. (3) |  |  |  |  |  |
| My data might be matched with other information about me, like records from my school. (5) |  |  |  |  |  |
| People using my data would have to do a special training course before they could access it. (6) |  |  |  |  |  |
| I would not be able to withdraw my data in the future. (9) |  |  |  |  |  |

Q2.11 When thinking about sharing **mental** health data, to what extent do you disagree or agree with the following statements?

|  | Strongly disagree (1) | Somewhat disagree (2) | Neither agree nor disagree (3) | Somewhat agree (4) | Strongly agree (5) |
| --- | --- | --- | --- | --- | --- |
| Mental health data should be used to understand more about mental illnesses. (1) |  |  |  |  |  |
| It should be impossible for mental health data to be linked back to the person who provided it. (2) |  |  |  |  |  |
| People should be asked for consent every time a researcher wants to use their data in a new project. (3) |  |  |  |  |  |
| People should have the right to opt out of mental health data sharing. (4) |  |  |  |  |  |
| I would be less likely to access NHS mental health services if I knew my data might be shared with researchers. (5) |  |  |  |  |  |
| It is important that mental health data is held by an organisation I trust. (6) |  |  |  |  |  |
| Researchers studying mental health should have advisors with personal experience of mental health conditions. (7) |  |  |  |  |  |

End of Block: Data sharing

Start of Block: Thank you next questions

Q3.1
Thank you for giving us your views on mental health data sharing. We would now like to ask you some questions about your mental and physical health. This information is really important as it will help us understand how people's health experiences shape the way they feel about people using their health data.
  
**If you are happy to continue to these questions, please select the arrow at the bottom right of the screen.**
 *Remember that if you want to skip any questions you can simply leave them blank.*

End of Block: Thank you next questions

Start of Block: Mental health

Q4.1

Q4.2
The following questions refer to your **mental**health only.

| Page Break |  |
| --- | --- |

Q4.3 How would you rate your mental health at the moment?

- Very poor (1)
- Poor (2)
- Average (3)
- Good (4)
- Very good (5)

Q4.4 Are you currently taking any prescribed medicines for a **mental** health condition?

- Yes (1)
- No (2)

Q4.5 Other than prescribed medication, are you currently receiving **NHS** treatment or support for a **mental** health condition?

- Yes (1)
- No (2)

Q4.6 Are you currently on one or more NHS waiting lists to receive treatment or support for a **mental** health condition?
 
If you are receiving treatment and you are also on a waiting list, please select 'Yes'.

- Yes (1)
- No (2)

| Page Break |  |
| --- | --- |

Q4.7 Have you personally experienced a mental health condition at any point during your life? 
 
We are interested in your experiences even if you have not received a formal diagnosis, and even if you are not experiencing symptoms at the moment. 
 
*Please note that we are referring to your personal mental health, not the mental health of your friends or family.*

- No, I have never had a mental health condition (1)
- Yes, I have or have previously had a mental health condition (2)
- Prefer not to say (3)

Skip To: End of Block If Have you personally experienced a mental health condition at any point during your life?    We ar... != Yes, I have or have previously had a mental health condition

Q4.8 Which mental health condition(s) have you personally lived with at any point during your life? 


Please read all the options and choose the one (or more) that best represents your mental health condition(s). If you have a mental health condition that is not on the list please select 'Other'.

- Addiction or substance use disorder (such as alcohol addiction, drug addiction) (9)
- Anxiety (such as generalised anxiety disorder, social anxiety, panic disorder, post-traumatic stress disorder) (2)
- Bipolar disorder (8)
- Body dysmorphic disorder (11)
- Depression (such as major depressive disorder, seasonal affective disorder, postnatal depression) (1)
- Eating disorder (such as anorexia, bulimia, binge eating disorder) (4)
- Obsessive-compulsive disorder (6)
- Personality disorder (such as borderline personality disorder, antisocial personality disorder) (7)
- Phobia (such as agoraphobia, phobia of spiders) (3)
- Schizophrenia or psychosis (5)
- Self-harm (12)
- Other (10)

Display This Question:

If Which mental health condition(s) have you personally lived with at any point during your life?  P... = Other

Q4.9 Please enter the name of this mental health condition:

________________________________________________________________

| Page Break |  |
| --- | --- |

Display This Question:

If If Which mental health condition(s) have you personally lived with at any point during your life?  P... q://QID31/SelectedChoicesCount Is Greater Than 1

Q4.10 Which do you consider to be your **primary** mental health condition? If you are unsure, choose the condition that has the biggest impact on your daily life at the moment (or, if you are currently well, the condition that used to have the biggest impact on your daily life). 
 
Please read all the options and choose the one that best represents your primary mental health condition. If your primary mental health condition is not on the list, please select 'Other'.

- Addiction or substance use disorder (such as alcohol addiction, drug addiction) (9)
- Anxiety (such as generalised anxiety disorder, social anxiety, panic disorder, post-traumatic stress disorder) (2)
- Bipolar disorder (8)
- Body dysmorphic disorder (11)
- Depression (such as major depressive disorder, seasonal affective disorder, postnatal depression) (1)
- Eating disorder (such as anorexia, bulimia, binge eating disorder) (4)
- Obsessive-compulsive disorder (6)
- Personality disorder (such as borderline personality disorder, antisocial personality disorder) (7)
- Phobia (such as agoraphobia, phobia of spiders) (3)
- Schizophrenia or psychosis (5)
- Self-harm (12)
- Other (10)

Display This Question:

If Which do you consider to be your primary mental health condition? If you are unsure, choose the c... = Other

Q4.11 Please enter the name of this mental health condition:

________________________________________________________________

| 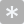 |
| --- |

Q4.12
In what calendar year (e.g. 2017, 1984) did you first have contact with the NHS regarding this mental health condition?
  
*If you have never had NHS contact about this condition, please enter 0.*

________________________________________________________________

Q4.13 Thinking back to that time, how satisfied were you with the **NHS** treatment/support you received for this mental health condition?

- Very dissatisfied (1)
- Dissatisfied (2)
- Neither satisfied nor dissatisfied (3)
- Satsified (4)
- Very satisfied (5)

| Page Break |  |
| --- | --- |

Q4.14

Q4.15
The following questions ask about your mental health during the last **12 months.**

| Page Break |  |
| --- | --- |

Q4.16
In the following question we are referring to the contact you have personally had with the NHS specifically. When responding to this question please ignore any treatment or support you have received from a non-NHS provider (such as a charity or private therapist). 
 
Please indicate whether you have had contact with the NHS during the last 12 months regarding any of the following (you can select more than one answer).

- I have approached the NHS regarding my mental health but have not received any treatment/support (1)
- Prescription medication (e.g. anti-depressant or anti-psychotic medicine) (2)
- One-to-one 'talking treatments' with an NHS therapist/psychologist/psychiatrist (3)
- Routine appointments with a psychiatrist, community psychiatric nurse (CPN) or equivalent (4)
- Group therapy provided by the NHS (5)
- Online resources provided by the NHS (e.g. SilverCloud) (6)
- NHS inpatient care (7)
- Crisis response/care (8)
- None of the above (9)

Display This Question:

If In the following question we are referring to the contact you have personally had with the NHS sp... = I have approached the NHS regarding my mental health but have not received any treatment/support

Or In the following question we are referring to the contact you have personally had with the NHS sp... = Prescription medication (e.g. anti-depressant or anti-psychotic medicine)

Or In the following question we are referring to the contact you have personally had with the NHS sp... = One-to-one 'talking treatments' with an NHS therapist/psychologist/psychiatrist

Or In the following question we are referring to the contact you have personally had with the NHS sp... = Routine appointments with a psychiatrist, community psychiatric nurse (CPN) or equivalent

Or In the following question we are referring to the contact you have personally had with the NHS sp... = Group therapy provided by the NHS

Or In the following question we are referring to the contact you have personally had with the NHS sp... = Online resources provided by the NHS (e.g. SilverCloud)

Or In the following question we are referring to the contact you have personally had with the NHS sp... = NHS inpatient care

Or In the following question we are referring to the contact you have personally had with the NHS sp... = Crisis response/care

Q4.17 Overall, how satisfied are you with the NHS support and treatment you have received for your **mental** health during the last **12 months?**

- Very dissatisfied (1)
- Dissatisfied (2)
- Neither satisfied nor dissatisfied (3)
- Satisfied (4)
- Extremely satisfied (5)

| Page Break |  |
| --- | --- |

Q4.18
Some people need to use the NHS many times during a short period of time, whilst other people only need to use the NHS occasionally.

 We would like you to think about how frequently you used the **NHS** for your **mental** health during the last **12 months**.

 *If the amount of times you used the NHS for mental health changed over the course of those 12 months, please answer in relation to the time you used the NHS most often.*

- I have not had mental healthcare from the NHS in the last 12 months (1)
- Once or twice in the last 12 months (2)
- About monthly (3)
- About once every 2 weeks (7)
- About weekly (4)
- More than once per week (5)
- I received NHS inpatient care for my mental health (6)

Q4.19 Please choose a number to indicate the extent to which you feel the symptoms of your mental health condition(s) are currently 'under control', from 1 (not at all under control) to 5 (fully under control).

- 1 (Not at all under control) (1)
- 2 (2)
- 3 (3)
- 4 (4)
- 5 (Fully under control) (5)

End of Block: Mental health

Start of Block: Physical health

Q5.1

Q5.2
The following questions refer to your **physical**health only.

| Page Break |  |
| --- | --- |

Q5.3 How would you rate your physical health at the moment?

- Very poor (1)
- Poor (2)
- Average (3)
- Good (4)
- Very good (5)

Q5.4 Are you currently taking any prescribed medicines for a physical health condition (please do not include contraceptives)?

- Yes (1)
- No (2)

Q5.5 Other than prescribed medications, are you currently receiving **NHS** support or treatment for a **physical** health condition?

- Yes (1)
- No (2)

Q5.6 Are you currently on one or more **NHS** waiting lists to receive treatment or support for a **physical** health condition?
 
If you are receiving treatment and you are also on a waiting list, please select 'Yes'.

- Yes (1)
- No (2)

| Page Break |  |
| --- | --- |

Q5.7 Do you have, or have you previously had, any physical disabilities or long-term **physical** health conditions?
 
*A disability or long-term physical health condition could include, but is not limited to, the following conditions: diabetes, arthritis, asthma, Crohn's disease, hypertension (high blood pressure), HIV/AIDS, multiple sclerosis, visual impairment.*

- Yes, more than one (1)
- Yes, one (2)
- No (3)
- Prefer not to say (4)

Skip To: Q5.13 If Do you have, or have you previously had, any physical disabilities or long-term physical health c... = No

Skip To: Q5.13 If Do you have, or have you previously had, any physical disabilities or long-term physical health c... = Prefer not to say

Display This Question:

If Do you have, or have you previously had, any physical disabilities or long-term physical health c... = Yes, more than one

Q5.8 In the following question we would like you to think about your **primary** long-term physical health condition. This means the condition that has the biggest impact on your daily life at the moment (or, if you are currently well, the condition that used to have the biggest impact on your daily life).

| 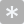 |
| --- |

Q5.9
In what calendar year (e.g. 2017, 1984) did you first have contact with the NHS regarding this long-term physical health condition?
  
*If you have never had NHS contact about this condition, please enter 0.*

________________________________________________________________

Q5.10 Thinking back to that time, how satisfied were you with the **NHS** treatment/support you received for this long-term physical health condition?

- Very dissatisfied (1)
- Dissatisfied (2)
- Neither satisfied nor dissatisfied (3)
- Satsified (4)
- Very satisfied (5)

| Page Break |  |
| --- | --- |

Q5.11 Please choose a number to indicate the extent to which you feel the symptoms of your long-term physical health condition(s) are currently 'under control', from 1 (not at all under control) to 5 (fully under control).

- 1 (Not at all under control) (1)
- 2 (2)
- 3 (3)
- 4 (4)
- 5 (Fully under control) (5)

| Page Break |  |
| --- | --- |

Q5.12 In the following section we want to know about your physical health in general (not just in relation to long-term physical health conditions).

| Page Break |  |
| --- | --- |

Q5.13
In the following question we are referring to the contact you have personally had with the NHS specifically. When responding to this question please ignore any treatment or support you have received from a non-NHS provider (such as private healthcare). 
 
Only select the options relating to your own healthcare (for example if you visited A & E with a relative/child but did not personally receive healthcare then leave 'An acute physical health condition which required hospital treatment' blank).
 
Please indicate whether you have had contact with the NHS during the last 12 months regarding any of the following (you can select more than one answer).

- I have approached the NHS regarding my physical health but have not received any treatment/support (1)
- Routine check up(s) not related to a diagnosed health condition (e.g. NHS health check, cervical screening, maternity care) (2)
- A long-term physical health condition (e.g. diabetes, high blood pressure) (3)
- A short-term physical health condition which required hospital treatment (e.g. broken leg, heart attack) (4)
- An short-term physical health condition which required a GP or other NHS health service (e.g. rash, persistent cough) (5)
- None of the above (6)

Display This Question:

If In the following question we are referring to the contact you have personally had with the NHS sp... = I have approached the NHS regarding my physical health but have not received any treatment/support

Or In the following question we are referring to the contact you have personally had with the NHS sp... = Routine check up(s) not related to a diagnosed health condition (e.g. NHS health check, cervical screening, maternity care)

Or In the following question we are referring to the contact you have personally had with the NHS sp... = A long-term physical health condition (e.g. diabetes, high blood pressure)

Or In the following question we are referring to the contact you have personally had with the NHS sp... = A short-term physical health condition which required hospital treatment (e.g. broken leg, heart attack)

Or In the following question we are referring to the contact you have personally had with the NHS sp... = An short-term physical health condition which required a GP or other NHS health service (e.g. rash, persistent cough)

Q5.14 Overall, how satisfied are you with the NHS support and treatment you have received for your **physical** health during the last **12 months?**

- Very dissatisfied (1)
- Dissatisfied (2)
- Neither satisfied nor dissatisfied (3)
- Satisfied (4)
- Very satisfied (5)

| Page Break |  |
| --- | --- |

Q5.15
Some people need to use the NHS many times during a short period of time, whilst other people only need to use the NHS occasionally.

 We would like you to think about how frequently you used the **NHS** for your **physical** health during the last **12 months**.

 *If the amount of times you used the NHS for physical health changed over the course of those 12 months, please answer in relation to the time you used the NHS most often.*

- I have not had physical healthcare from the NHS in the last 12 months (1)
- Once or twice in the last 12 months (2)
- About monthly (3)
- About once every 2 weeks (7)
- About weekly (4)
- More than once per week (5)
- I received NHS inpatient care for my physical health (6)

End of Block: Physical health

Start of Block: Demographics

Q6.1 What is your gender?

- Male (1)
- Female (2)
- Non-binary (3)
- Prefer to self-describe (4)
- Prefer not to say (5)

Display This Question:

If What is your gender? = Prefer to self-describe

Q6.2 Please specify:

________________________________________________________________

| 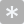 |
| --- |

Q6.3 What is your age (in years)?

________________________________________________________________

| Page Break |  |
| --- | --- |

Q6.4

Q6.5 Which country do you currently live in? 


If you spend time in more than one country, please select the country in which you access or would be more likely to access NHS healthcare.

- England (1)
- Scotland (2)
- Wales (3)
- Northern Ireland (4)
- Outside the UK (5)

Q6.6 How would you describe your national identity?

- English (1)
- Welsh (2)
- Scottish (3)
- Northern Irish (4)
- British (5)
- Other (6)

Display This Question:

If How would you describe your national identity? = Other

Q6.7 Please specify:

________________________________________________________________

Q6.8 What is your ethnic group?

- White (1)
- Mixed/Multiple ethnic groups (2)
- Asian/Asian British (3)
- Black/African/Caribbean/Black British (4)
- Other ethnic group (5)

Q6.9 What is the highest level of education you have completed?

- Primary school (1)
- GCSE or equivalent (e.g. O-level, CSE) (2)
- A-level or equivalent (3)
- Vocational or college qualification (e.g. NVQ, HND) (4)
- Undergraduate degree (5)
- Postgraduate degree or professional qualification (e.g. Master's degree, PhD, PGCE) (6)

End of Block: Demographics

Start of Block: Contact and withdrawal

| 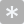 |
| --- |

Q7.1 Thank you for your responses to this survey. For the next stage of our research, we will be conducting telephone/Skype interviews with people who have experience of living with one or more mental health conditions. We anticipate that the interviews will take place in early to mid 2019.

 If you would be interested in taking part in an interview, please enter your email below.

 *By entering your email address you are only expressing an interest; you will be able to decide nearer the time whether or not you want to take part in an interview. Please be aware that we probably won't be able to interview everyone who volunteers.*

________________________________________________________________

| Page Break |  |
| --- | --- |
