# Supplementary material for: Experience of clinical services shapes attitudes to mental health data sharing: findings from a UK-wide survey
Source: BMC Public Health. 2022 Feb 19;22:357. doi: 10.1186/s12889-022-12694-z (PMC8858475; doi:10.1186/s12889-022-12694-z)
Supplement: Supplementary file 2 — Additional file 2. [file 12889_2022_12694_MOESM2_ESM.docx]

**Supplementary material 2:** Ordinal logistic regression model examining the effect of demographic factors on likelihood of sharing physical health data – adapted analysis

The corresponding analysis in the text did not meet the assumption of proportional odds. Examination of binomial logistic regressions suggested that the problem may lie in the mental illness (ever) variable.

As such, the “prefer not to say” option was removed from the mental illness (ever) variable and the analysis was re-run.

A cumulative odds ordinal logistic regression with proportional odds was run to determine the effect of gender, age, ethnicity, location, education, long-term physical illness (ever), mental illness (ever) [without “prefer not to say”], self-rating of current physical health and self-rating of current mental health on willingness to share physical health data. Being paid for participation in the survey was included as a co-variate. The final model statistically significantly predicted likelihood of sharing physical health data over and above the intercept-only model, χ2(23) = 58.061, p < .001. The assumption of proportional odds was met (χ2(69) = 70.964, p = .412). Level of education had a marginally significant effect on the prediction of willingness to share health data, χ2(5) = 10.23, p = .069. The odds of people whose highest level of education was a postgraduate degree being willing to share their physical health data were 1.64 (95% CI, 1.21 to 2.22) times that of people whose highest level of education was a vocational or college level qualification, (Wald χ2(1) = 10.03, p = .002). Without the “prefer not to say” option, the variable measuring experience of mental illness had no significant effect on willingness to share physical health data. An increase in self-rated physical health was associated with an increase in the odds of sharing physical health data, with an odds ratio of 1.142 (95% CI, 1.019 to 1.279), Wald χ2(1) = 5.230, p = .022. An increase in self-rated mental health was also associated with an increase in the odds of sharing physical health data, with an odds ratio of 1.169 (95% CI, 1.046, 1.305), Wald χ2(1) = 7.653, p = .006.
